# Race and nodal disease status are prognostic factors in patients with stage IVB cervical cancer

**DOI:** 10.18632/oncotarget.25962

**Published:** 2018-08-17

**Authors:** Shin Nishio, Koji Matsuo, Koji Yonemoto, Mototsugu Shimokawa, Masayuki Hosaka, Michiko Kodama, Takahito M. Miyake, Kimio Ushijima, Toshiharu Kamura, Shannon N. Westin, Pamela T. Soliman, Robert L. Coleman

**Affiliations:** ^1^ Department of Gynecologic Oncology and Reproductive Medicine, University of Texas, MD Anderson Cancer Center, Houston, TX, USA; ^2^ Department of Obstetrics and Gynecology, Kurume University School of Medicine, Kurume, Fukuoka, Japan; ^3^ Division of Gynecologic Oncology, Department of Obstetrics and Gynecology, University of Southern California, Los Angeles, CA, USA; ^4^ Norris Comprehensive Cancer Center, University of Southern California, Los Angeles, CA, USA; ^5^ Biostatistics Center, Kurume University, Kurume, Fukuoka, Japan; ^6^ Clinical Research Institute, National Kyushu Cancer Center, Fukuoka, Japan

**Keywords:** stage IVB, cervical cancer, prognostic factor, African-American, para-aortic chain

## Abstract

**Background:**

Patients presenting with stage IVB cervical cancer pose a significant clinical challenge. While previous studies described several poor prognostic factors, they were limited by small sample sizes. The aim of this study was to identify clinicopathological prognostic factors in a large sample of patients with stage IVB cervical cancer at a single institution.

**Methods:**

Patients with primary stage IVB cervical cancer diagnosed between 1992 and 2011 were extracted from a search of the MD Anderson Cancer Center registry. Clinicopathological data retrieved from their medical records included demographics (age and race), tumor characteristics (primary lesion size, grade, and histology), TNM classification, and metastatic site (nodal/organ). Treatment approach (radiation, chemotherapy, or both) and intent (palliation or curative) were recorded. Survival rates were evaluated using the Kaplan-Meier method. Cox proportional hazards regression was used to model the association between key variables and overall survival (OS).

**Results:**

Two hundred sixty-six patients with stage IVB cervical cancer were identified. Their median OS was 12.7 months. The hazard ratio for African-Americans vs. patients with other ethnicities was 1.76 (95% confidence interval [CI], 1.18–2.54, *P* = 0.0063), and that for patients with para-aortic nodes alone vs. more extensive metastases was 0.37 (95% CI, 0.26–0.51, *P* < 0.0001). Other clinicopathological factors were not significantly associated with survival.

**Conclusions:**

African-American race was an independent adverse prognostic factor in this cohort. On the other hand, nodal disease in the para-aortic chain alone predicted a favorable prognosis.

## INTRODUCTION

Cervical cancer is the second most common type of cancer afflicting women worldwide, and has an annual incidence of 530,000 new cases [1]. Despite recent advances in surgery and radiotherapy, this disease is responsible for approximately 250,000 deaths globally each year [1]. Roughly 10% of patients with newly diagnosed cervical cancer have distant metastasis at the time of disease detection and are thus diagnosed with International Federation of Gynecology and Obstetrics (FIGO) stage IVB disease. These patients represent a heterogeneous population ranging from patients with nodal metastasis to those with metastasis to the visceral organs; however, they collectively have a poor prognosis. According to information maintained in the U.S. Surveillance, Epidemiology and End Results (SEER) database, the 5-year survival rate for patients with distant disease is only 15%, which is significantly lower than the 91% and 57% rates observed in patients with localized disease or regional metastasis, respectively [2]. Because of the heterogeneity of these patients' signs and symptoms at presentation, the type of treatment varies according to each patient's condition, performance status, and disease characteristics, as well as the physician's assessment of treatment impact. Historically, patients with metastatic cervical cancer have only received palliative treatment. While no particular treatment has been demonstrated to be superior, a variety of therapeutic options are currently available: patients with limited distant nodal metastasis, such as metastasis to the para-aortic or supraclavicular lymph nodes, might be candidates for radiotherapy with curative intent involving an extended radiation field [3]. Platinum-based combination chemotherapy or palliative radiotherapy is generally recommended for patients who are not candidates for definitive radiotherapy [4, 5]. The prognostic factors for patients with stage IVB cervical cancer have been investigated in several studies; metastatic site [6], poor performance status [7], and non-squamous cell carcinoma histology [8] have been reported as significant predictors of survival.

Several small retrospective studies in 50–100 patients have been performed in Asia, mainly Japan and Korea [7–13]. To our knowledge, however, studies including other ethnic groups (i.e., Caucasians and African-Americans) have not been performed. Therefore, we retrospectively investigated a large number of patients with stage IVB cervical cancer who belonged to different ethnic groups.

## RESULTS

**Figure 1:**
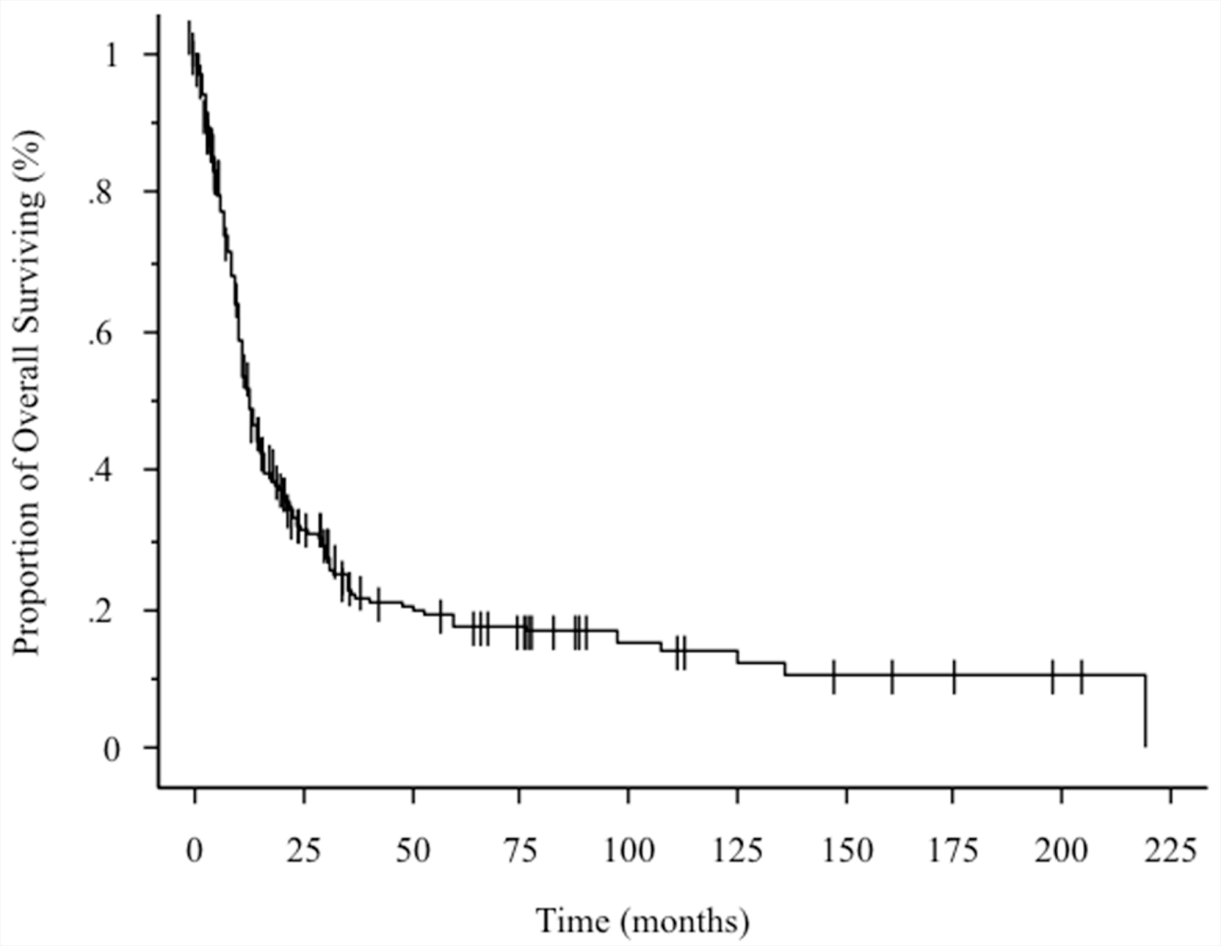
Kaplan-Meier curve showing overall survival in 266 patients with stage IVB cervical cancer

Two hundred sixty-six patients were treated at our institution between 1992 and 2011; their characteristics are shown in Table 1. Their median age was 50 years (range 27–86 years). In terms of race, 56.4% were Caucasians, 16.5% were African-American, 22.9% were Hispanic, 2.6% were Asian, and 1.6% were other ethnicities. The median primary tumor diameter was 6 cm; the maximum was 16 cm. Histopathologically, 69.9% of the patients had squamous cell carcinomas, 18.8% had adenocarcinomas, 6.4% had neuroendocrine tumors, and 4.9% had unclassified tumors. Hydronephrosis was noted in 89 patients, and was bilateral in 39. The metastatic lesion sites included the main organs in 132 patients and the lymph nodes in 221 (with overlap). Among the main organs, the lung was the most common site of metastasis (60 patients), followed by the liver (30 patients). Intraperitoneal cavity and para-aortic lymph node metastases were detected in 138 patients (the largest subgroup of lymph node metastases). Of these patients, 117 of them underwent CT-guided biopsy, and 21 patients underwent laparoscopic lymph-node dissection because they participated in a clinical study of retroperitoneal lymph-node dissection; para-aortic lymph node metastases alone were detected in 91 patients. As for the extraperitoneal region, 39 and 26 patients had supraclavicular and inguinal lymph node metastases, respectively. The median overall survival (OS) was 12.7 months (range: 0.5–219.1 months) (Figure 1). The follow-up time period was calculated from the first visit day to last contact day. The median follow-up time was 18.1 months.

**Table 1:** Patient characteristics (n=266)

Factor	No. (%)
**Age, years, median (range)**	50 (27–86)
**Race**	
Caucasian	150 (56.4)
African-American	44 (16.5)
Hispanic	61 (22.9)
Asian	7 (2.6)
Others	4 (1.6)
**Tumor diameter (cm), median (range)**	6 (2-16)
**Histology**	
Squamous cell carcinoma	186 (69.9)
Adenocarcinoma	50 (18.8)
Neuroendocrine	17 (6.4)
Unclassified	13 (4.9)
**Differentiation**	
Well	15 (5.6)
Moderate	67 (25.2)
Poorly	137 (51.5)
Unclassified	47 (17.7)
**Hydronephrosis**	
Right	30(11.3)
Left	20(8.8)
Bilateral	39(14.7)
None	177(65.2)
**Clinical T stage (FIGO 1988)**	
IB1	9 (3.4)
IB2	48 (18)
IIA	16 (6)
IIB	55 (20.7)
IIIA	13 (4.9)
IIIB	103 (38.7)
IVA	22 (8.3)
Metastatic site^*^	
**Organ**	**132**
Brain	1
Lung	60
Liver	30
Spleen	2
Abdominal wall	8
Navel	2
Omentum	12
Vulva	2
Bone	15
**Lymph node**	**221**
Neck	10
Supraclavicular	39
Mediastinum	4
Axilla	4
Intraperitoneal/para-aortic (para-aortic alone)	138 (91)
Inguinal	26

^*^Duplicated cases included.

FIGO, International Federation of Gynecology and Obstetrics.

The initial treatments undergone by the patients are summarized in Table 2. Of the 266 patients, 6 (2.3%) initially underwent surgery while 181 (68%) initially received radiotherapy; 72 patients in the latter group (39.8%) received radiotherapy alone while 109 (61.2%) underwent chemoradiotherapy. Furthermore, 72 patients initially received chemotherapy, wherein carboplatin plus paclitaxel was the most common regimen (56.9%). Seven patients received best supportive care.

**Table 2:** Initial treatment (n=266)

Treatment	No. (%)
**Surgery**	
Hysterectomy+pelvic lymphadenectomy	**6 (2.3)**
**Radiotherapy**	**181 (68)**
Radiotherapy alone	72
Concurrent chemoradiotherapy	109
**Chemotherapy**	**72 (27.1)**
Carboplatin/paclitaxel	41
Cisplatin/topotecan	6
Cisplatin/etoposide	5
Cisplatin/pemetrexed	4
Cisplatin/paclitaxel	3
Cisplatin/gemcitabine	2
Cisplatin	1
Paclitaxel/topotecan	3
Paclitaxel/bevacizumab	3
Topotecan	1
Docetaxel	1
Regorafenib	1
Cediranib	1
**Best supportive care**	**7 (2.6)**

Table 3 shows the factors that affect OS as identified via univariate and multivariate Cox regression model analyses. Variables associated with poorer OS on univariate analysis included clinical T stage III/IV and para-aortic lymph node metastasis alone. In contrast, age, African-American race, tumor diameter, poorly differentiated lesions, histologic type and the presence of hydronephrosis were unrelated to survival.

**Table 3:** Cox regression analysis of factors influencing overall survival

Factor	Univariate analysis			Multivariate analysis		
	Hazard Ratio	95% CI	*P*-value	Hazard Ratio	95% CI	*P*-value
**Age (years): ≥50 (vs. <50)**	0.96	0.73–1.27	0.7841	0.79	0.59-1.08	0.7893
**Race: African-American (vs. non- African-American)**	1.33	0.93–1.95	0.1176	**1.76**	**1.18–2.54**	**0.0063**
**Tumor diameter: ≥4 cm (vs. < 4 cm)**	1.36	0.76–2.45	0.3002	0.88	0.49-1.74	0.6968
**Differentiation: poorly (vs. non-poorly)**	1.15	0.87–1.53	0.3212	1.04	0.78-1.40	0.7893
**Histology: Squamous cell carcinoma (vs. non-squamous cell carcinoma)**	0.83	0.62–1.12	0.2248	0.90	0.69-1.32	0.7414
**Hydronephrosis: Present (vs. absent)**	1.29	0.95–1.73	0.0996	1.02	0.70–1.48	0.9128
**Clinical T stage: stages III, IV (vs. stages I, II)**	**1.42**	**1.07–1.88**	**0.0144**	1.35	0.94–1.90	0.1036
**Metastatic site: Para-aortic lymph node alone (vs. other sites)**	**0.38**	**0.28–0.52**	**<0.0001**	**0.37**	**0.26–0.51**	**0.0001**

CI, confidence interval.

After adjusting for the abovementioned variables, multivariate analysis showed that African-American race was significantly associated with poorer OS, while para-aortic lymph node metastasis alone was associated with improved OS. Hydronephrosis and clinical T stage did not influence OS.

## DISCUSSION

To our knowledge, this study to identify prognostic factors in patients with stage IVB cervical cancer included the largest cohort to date. Our results showed that African-American race and para-aortic node metastasis alone were predictors of poor and favorable OS in patients with stage IVB cervical cancer, respectively. Previously identified prognostic factors for stage IVB cervical cancer include performance status, age, histological subtype, main organ metastasis, and distant metastasis [7–13].

For most types of cancer, American-African heritage is associated with the highest mortality rates and worst survival outcomes of any population [14]; this observation has been a concern for nearly 40 years [15]. Although it is incontrovertible that racial disparities exist in the incidence of cancer and cancer-related mortality, the reasons for this are not clear and remain controversial. Some investigators have suggested that biological differences among distinct ethnic groups cause different levels of susceptibility to disease [16, 17], while others posited that socioeconomic and cultural influences are also impactful [18].

However, it is less clear why African-American ethnicity was a negative predictive factor in our study. African-Americans may have limited access to care for comorbidities not reflected in performance status, or may have biologically worse cervical cancers [19–23]. It was previously reported that race is not an independent predictor of survival for patients with cervical cancer in an equal access environment such as the military [24]. Interestingly, however, a Gynecologic Oncology Group ancillary data study in women with recurrent cervical cancer found that cisplatin-based chemotherapy was better tolerated by African-American women [25]. All previous studies of stage IVB cervical cancer have been based on data obtained from Asians, including Japanese and Koreans [7–13]. Therefore, our results ought to be considered novel findings and suggest that ethnicity should be adjusted for in future clinical studies.

**Figure 2:**
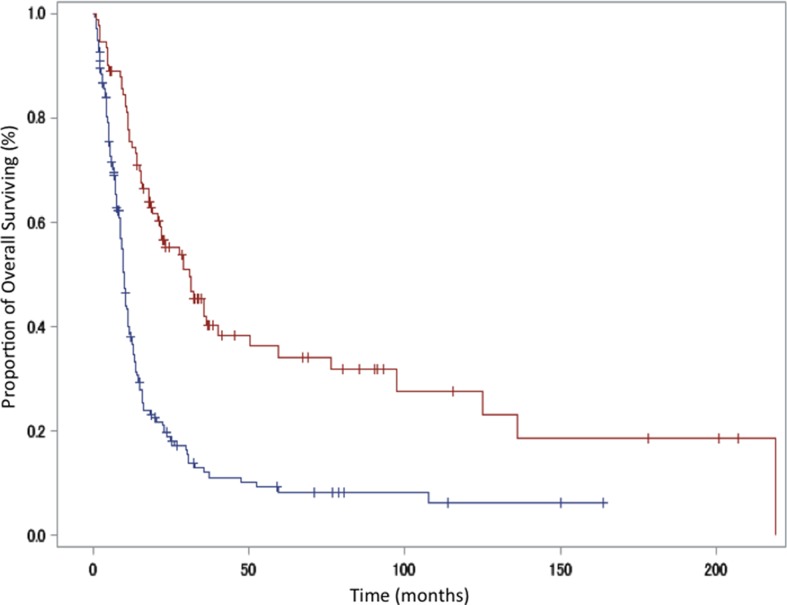
Kaplan-Meier curve showing overall survival in patients with para-aortic lymph-node metastasis versus those with other metastases Red line, para-aortic lymph-node metastasis group; blue line, other metastases group.

Our analysis also showed that para-aortic lymph node metastasis alone was a significant predictor of favorable OS. This observation was consistent with previous studies that showed that patients with stage IVB cervical cancer who had metastases confined to lymph nodes were previously shown to have superior survival rates than those with distant organ metastases [7–13]. The FIGO classification of cervical cancer was revised in 2009 [26], however, stage IVB cervical cancer remains defined as tumor extension beyond the true pelvis. M1 is defined as distant metastasis (including peritoneal spread; involvement of supraclavicular, mediastinal, or para-aortic lymph nodes; and lung, liver, or bone involvement). Para-aortic lymph node metastases and those of other types also fall under the same staging category. In our study, the median survival time was 22.1 months in patients with para-aortic lymph-node metastasis alone compared to 9.0 months in patients with other metastases (Table 4 and Figure 2). Multivariate analysis also showed that outcomes significantly differed between these subgroups of patients, with a hazard ratio of 0.37. Clinically, the rationale for including all these different types of metastases under “stage IVB” cervical cancer is questionable; it may be more logical to reclassify diseases with para-aortic lymph node metastases as stage IVB-1 and those with other types of metastases as stage IVB-2. In fact, in a prospective study of patients with stage IVB cervical cancer, the inclusion criteria were stated as the presence of “at least 1 metastatic lesion outside the pelvic cavity except in the para-aortic and/or inguinal lymph nodes” [27].

**Table 4:** Difference in initial treatment

Treatment	Para-aortic lymph node metastasis (n=91)	Other types of metastasis (n=175)
	**No. (%)**	**No. (%)**
**Surgery**	**3 (3.3)**	**3 (1.7)**
**Radiotherapy**	**86 (94.5)**	**95 (54.2)**
Radiotherapy alone	30	42
Concurrent chemo-radiotherapy	56	53
**Chemotherapy**	**1 (1.1)**	**71 (40.6)**
**Best Supportive Care**	**1 (1.1)**	**6 (3.5)**

A strength of our study is that we were able to enroll a relatively large number of patients with stage IVB cancer who were treated at a single institution, including non-Asian patients. The presence of metastases in para-aortic lymph nodes was confirmed histologically in all patients (of note, patients with positive aspiration cytology results alone not included). The growing popularity of positron emission tomography (PET) and computed tomography (CT) has facilitated the detection of para-aortic lymph node metastases. However, a certain number of false-positive cases were detected on conventional CT, potentially leading to overdiagnosis [28].

A major limitation of our study was that it was retrospective and performed over a relatively long period of time. A total of 334 patients were initially identified, but 68 patients were excluded because they lacked sufficient data in their medical records; therefore, selection bias cannot be ruled out. Furthermore, the modes of radiotherapy as well as the chemotherapy regimens were changed during the study period. Lifestyle and socioeconomic factors are important prognostic factors in patients with cervical cancer [29, 30]. Although we could not evaluate these factors in the present study, the results may have changed if these factors had been included in the analysis. We focused only on OS; however, relief and supportive care are also very important considerations for these patients' quality of life. To eliminate these potential biases, prospective multi-institutional investigations ought to be conducted.

In conclusion, African-American ethnicity and para-aortic lymph node metastasis alone are significant prognostic indicators for patients with stage IVB cervical cancer.

## MATERIALS AND METHODS

### Patients

After receiving the approval of the University of Texas MD Anderson Cancer Center institutional review board, data were abstracted from a database of patients with cervical cancer treated at the institution definitively between January 1, 1992 through December 31, 2011.

**Figure 3:**
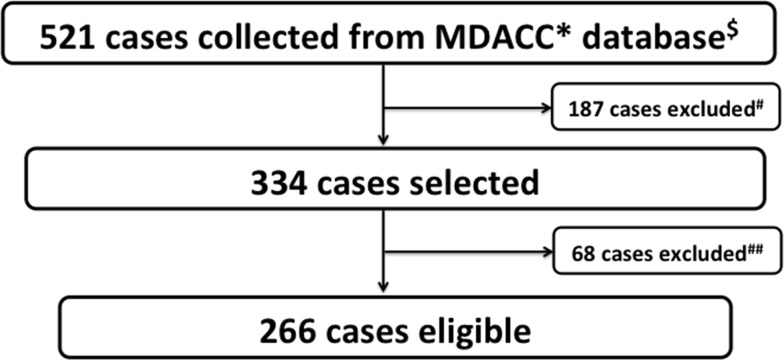
Study selection schema ^*^MDACC: MD Anderson Cancer Center. ^$^Key word: metastatic × cervical cancer. ^#^Non-stage IVB (e.g. recurrent cases). ^##^Incomplete medical records.

A search of the MD Anderson Cancer Center database using the keywords “metastatic” and “cervical cancer” identified 521 patients. After applying our exclusion criteria, 266 patients were ultimately included in our analysis (Figure 3).

All patients underwent at least a cervical biopsy, full physical examination, pelvic examination, routine laboratory tests, and evaluation for metastasis involving chest radiography and CT of the abdomen and pelvis. Stage IVB disease was defined as primary cervical cancer combined with distant lymphatic spread beyond the pelvis according to the FIGO staging system for cancer of the uterine cervix. Distant metastasis was confirmed by imaging studies including CT and PET/CT or by fine-needle aspiration biopsy. All cases of lymph node metastasis were confirmed histologically; tumor samples were reviewed by one or more pathologists with specific expertise in gynecologic malignancies.

### Treatment

Therapeutic strategies were selected on an individual basis. Some patients underwent total hysterectomy (some underwent radical hysterectomy) and bilateral salpingo-oophorectomy; moreover, some patients underwent pelvic and/or para-aortic lymphadenectomy. For radiotherapy, the abdominopelvic field encompassed a volume that included the primary mass, entire uterus, paracervical, parametrial, and uterosacral regions and the external iliac, hypogastric, obturator, and para-aortic nodes. The superior border of the abdominopelvic field was usually the T12–L1 interface, but was adjusted based on the position of the positive para-aortic nodes. The median daily fraction of external beam radiation therapy (EBRT) was 1.8 Gy (range, 1.5–2.0 Gy) administered once daily, 5 times per week using 3D concurrent chemoradiotherapy or intensity-modulated radiotherapy with a linear accelerator or a helical tomotherapy system. Chemotherapy regimens included cisplatin (40 mg/m^2^) weekly or 5-fluorouracil (1,000 mg/m^2^) plus cisplatin (50 mg/m^2^) monthly. The median dose was 59.4 Gy to the para-aortic nodes and 50.4 Gy to the entire pelvis. After completing EBRT, high-dose-rate intracavitary brachytherapy was administered using Fletcher-Suit after loading applicators. Six to seven fractions of 4–5 Gy were delivered to point A two–three times per week, with no EBRT treatment on the same day as brachytherapy. Parametrial boosts were integrated between brachytherapy sessions.

Additionally, some patients eligible for chemotherapy participated in a clinical study; the chemotherapeutic regimens employed included carboplatin-based or cisplatin-based doublets. Best supportive care was defined as treatment targeting the relief of symptoms without surgery, radiotherapy, or chemotherapy.

### Statistical analysis

Univariate and multivariate Cox regression models were used to evaluate factors previously described as prognostic in patients with cervical cancer [7–13]. These included age: ≥50 years (vs. <50 years); race: African-American (vs. non-African-American); tumor diameter: ≥4 cm (vs. <4 cm); differentiation: poorly differentiated (vs. non-poorly differentiated); histologic type: squamous cell carcinoma (vs. non-squamous cell carcinoma); hydronephrosis: present (vs. absent); clinical T stage: stage III–IV (vs. stage I–II); and metastatic site: para-aortic lymph nodes alone (vs. other sites). OS was estimated using the Kaplan-Meier method; the log-rank test was used to compare differences between survival curves. All *P*-values were 2-sided; *P*-values, <0.05 were considered statistically significant. All analyses were performed using the SAS software, release 9.1 (SAS Institute, Cary, NC, USA).
